# A Clinical Phrase Book &c.

**Published:** 1853-05

**Authors:** 


*A Clinical Phrase Book in English and German*, containing the usnal questions and answers employed in examining and prescribing for patients, questions in asking for and buying medicines, &c., with an English-German and German-English Pronouncing Lexicon of all the words occurring in the phrases, with the chief technical terms of Medical Writers and Apothecaries; Grammatical Appendix, Table of idioms, &c., designed to aid physicians and surgeons in hospitals, almshouses, and private practice; also druggists and pharmaceutists in dispensing their prescriptions. By MONTGOMERY JONES, M.D.

THE above is the long title to a small book of 308 pages, 12mo, published by Lindsay & Blackiston, Philadelphia. The object of the work is explained on the title page, and we have no fault to find with the manner in which the author has accomplished his task. It is not *designed* as a substitute for more full and complete works; and yet we fear, that this unpretending volume, in the hands of many, would divert the attention from those works, where only a *useful* knowledge of the language can be obtained—we say useful, for we are satisfied that a language can be acquired only by long and laborious study, and with a partial knowledge, misunderstandings are liable to occur between the physician and his patient, leading, sometimes, to very serious mistakes in prescribing. If, however, a larger and more complete work be used as a text book, and the phrase book resorted as an aid in cases of emergency, there can be no objection to it. Indeed, it may sometimes be very useful. J.

